# Intra- and Postoperative Botulinum Toxin Injection in Postsurgical Pain Management: A Literature Review

**DOI:** 10.1155/prm/6649252

**Published:** 2025-11-30

**Authors:** Sogol Alikarami, Saereh Hosseindoost, Ahmad Reza Dehpour, Zahra Rezaei, Hossein Majedi

**Affiliations:** ^1^Pain Research Center, Neuroscience Institute, Imam Khomeini Hospital Complex, Tehran University of Medical Sciences, Tehran, Iran; ^2^Brain and Spinal Cord Injury Research Center, Neuroscience Institute, Tehran University of Medical Sciences, Tehran, Iran; ^3^Department of Pharmacology, School of Medicine, Tehran University of Medical Sciences, Tehran, Iran; ^4^Experimental Medicine Research Center, Tehran University of Medical Sciences, Tehran, Iran; ^5^Department of Medicinal Chemistry, Faculty of Pharmacy, Tehran University of Medical Sciences, Tehran, Iran

**Keywords:** analgesics, botulinum neurotoxin, pain, postoperative pain

## Abstract

Postoperative pain remains a significant challenge in surgical services, which necessitates improving analgesic strategies to enhance patient outcomes. Botulinum neurotoxin (BoNT), which was primarily approved for the treatment of strabismus and blepharospasm, has demonstrated a promising impact on pain reduction through mechanisms such as neurotransmitter inhibition, receptor modulation, glial activity suppression, and interactions with opioidergic and GABAergic systems. A number of studies have investigated BoNT's impact on postsurgical pain. However, there is a lack of evaluation of its efficacy, safety, and optimal administration protocols across different surgical settings. This study aims to provide a comprehensive overview of the existing literature on the efficacy and complications of intra- and postoperative BoNT injections in managing postsurgical pain across various surgical procedures, including orthopedic and head and neck surgeries, mastectomy, hemorrhoidectomy, and fissurectomy.

## 1. Introduction

Postoperative pain is a significant challenge in surgical care. Each year in the USA, more than 70 million patients undergo surgery, and 4 out of 5 of them experience acute postoperative pain [1]. If the pain is not managed, it can lead to longer hospital stays and secondary complications such as deep vein thrombosis, postsurgical infections, and prolonged recovery [2, 3]. Moreover, inadequate control of acute pain contributes to the development of chronic postoperative pain (CPSP) [4, 5]. This condition affects 5%–58% of individuals who undergo surgical procedures and imposes significant costs on both patients and the healthcare system [6, 7]. These challenges emphasize the crucial need for minimally invasive techniques and early postsurgical pain management with an interdisciplinary approach to prevent further negative consequences [8, 9].

Opioids and nonopioid analgesics, such as nonsteroidal anti-inflammatory drugs (NSAIDs), form the cornerstones of postoperative pain management. However, excessive use of opioids can result in various adverse effects, such as respiratory depression, gastrointestinal disturbances, and cognitive impairments [10–12]. Additionally, opioid-induced tolerance, paradoxical hyperalgesia, and physical dependence can complicate pain management [11, 13, 14]. While NSAIDs are effective for relieving pain and inflammation, they also have potential side effects, including gastrointestinal and cardiovascular issues, renal impairments, and central nervous system effects [15]. NSAIDs also increase the risk of pseudarthrosis and impaired bone healing when used early after spinal fusions [16]. Local approaches, such as regional blocks and local infiltration analgesia, mitigate pain while minimizing the risk of analgesic-related side effects. Integrating these methods with systemic analgesics can further enhance pain control [17–19].

Botulinum neurotoxin (BoNT) is commonly used for conditions such as overactive bladder, strabismus, blepharospasm, cervical dystonia, hyperhidrosis, and extremity spasticity. BoNT has also been indicated for the prophylactic management of chronic migraine [20]. Beyond its approved applications, its potential for pain treatment is increasingly recognized [21]. BoNT has demonstrated effectiveness in reducing pain through several biochemical processes, including inhibition of neurotransmitter release, regulation of pain-related receptors, suppression of glial activity, and interaction with opioidergic and GABAergic systems [22, 23]. BoNT's analgesic effect has been investigated in conditions such as spinal cord injuries, diabetic neuropathy, myofascial syndrome, headaches, and pelvic pain syndrome [24–26]. Moreover, when administered during or after surgeries such as hemorrhoidectomy, mastectomy, and limb lengthening surgeries, BoNT has demonstrated promising results in the prevention and treatment of postsurgical pain [27–29]. This review aims to evaluate the existing literature on the intra- and postoperative application of BoNT in the management of postsurgical pain.

## 2. Materials and Methods

We searched the PubMed database on January 13, 2025, using the appropriate keywords, “postoperative pain” and “botulinum neurotoxin”. The detailed search strategy in PubMed is presented in Supporting Table 1. Inclusion criteria were original articles in English that focused on the intra- or postoperative use of BoNT in the management of postoperative pain. We applied no publication date limits to our search.

Nonhuman and non-English studies, review articles, abstracts, protocols, letters to the editor, conference papers, case reports, or case series with fewer than 10 participants were excluded. In addition, studies addressing preoperative BoNT application for postsurgical pain were also removed.

Additionally, we performed a manual search of the most relevant search results from Google Scholar and citations of related articles to ensure no appropriate studies were overlooked. All the obtained articles were imported into the EndNote library. The studies were screened by reviewing their titles and abstracts. Subsequently, the full text of the remaining articles was examined to select eligible articles. The data from the included studies were extracted and recorded on the pre-prepared table.

## 3. Results

### 3.1. Overview of Study Characteristics

**Figure 1:**
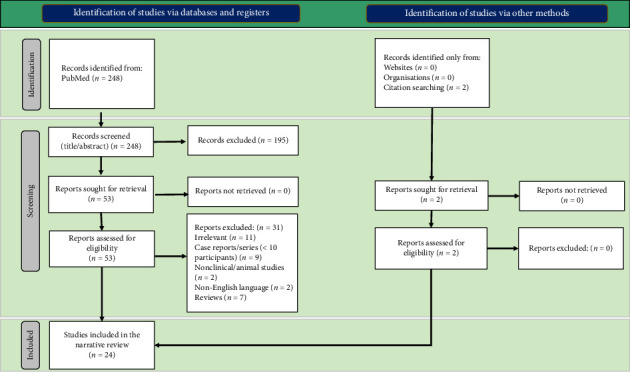
Flow diagram demonstrating the identification, screening, and inclusion process of studies.

Figure 1 shows the detailed screening process. Initial searching of the PubMed database yielded 248 articles. Of these, 195 studies were excluded during the title and abstract screening. A total of 53 studies were screened in full text. From this group, 31 reports were excluded because they were irrelevant, not in English, were case reports or case series with fewer than 10 participants, review articles, or nonclinical studies. Two extra articles were added from a manual search. Ultimately, a total of 24 articles were included [27, 29–51].

Most of the included studies were randomized clinical trials (RCT) [30, 31, 33–39, 46–52], followed by retrospective [40, 41, 43, 44], pilot studies [32, 42, 45], and a nonrandomized prospective clinical trial [29]. Sample sizes ranged from 8 to 281 participants. BoNT's efficacy was investigated in various surgical specialties, including orthopedic, gastrointestinal, mastectomy and reconstruction, head and neck, and urological surgeries. In 19 articles, the BoNT was injected intraoperatively, while 5 of them followed postoperative injection. Pain intensity was commonly assessed using the visual analog scale (VAS). The interventions differed in BoNT dosage (ranging from 10 to 400 U), injection methods (intramuscular, subcutaneous, intraarticular, or intersphincteric), and timing (intra- or postoperative). Comparators typically included placebo (saline injections).  Table 1 indicates the characteristics of the included studies.

### 3.2. Pain Intensity

#### 3.2.1. Orthopedic Surgeries

Table 2 summarizes the results of the included studies on the effect of BoNT in postoperative pain reduction and analgesic requirement. In orthopedic surgeries, the efficacy of intraoperative BoNT for postoperative pain management differed depending on the procedure. For limb lengthening, an RCT of 125 patients showed significantly lower maximum pain on the first day following surgery in the BoNT group compared to the placebo group, which suggests the potential of BoNT in immediate postoperative pain reduction; however, the analgesic effect did not extend to days 2–4 [35].

Two other trials reported no significant differences in pain relief between the BoNT and placebo groups at any follow-up time points, ranging from 4 to 48 weeks [34, 36]. [NO_PRINTED_FORM] These findings show that although BoNT may provide immediate pain control, its efficacy for long-term pain relief after limb-lengthening surgeries remains uncertain.

Regarding total knee arthroplasty, an RCT reported that a significantly greater proportion of patients in the BoNT group reported substantial pain improvement (VAS score reduction of ≥ 2 points) at 2 months compared to the placebo group. This difference in the proportion of responders remained significant across all of the three time points of 2, 3, and 4 months. The BoNT group also experienced a significantly longer duration of pain relief [33]. These findings suggest that BoNT may be effective for managing postoperative pain after large joint surgeries, such as total knee arthroplasty, and its effect can persist for at least 4 months after the surgery.

#### 3.2.2. Gastrointestinal Surgeries

BoNT has provided significant pain relief in managing pain following hemorrhoidectomy, with significant pain reduction from the immediate postoperative period through the first 2 weeks.

Multiple RCTs confirm that BoNT significantly reduced pain within the first 24 h following hemorrhoidectomy [46, 48]. One study focusing on Ferguson's closed technique hemorrhoidectomy reported significantly lower pain scores in the BoNT group compared to the control group at both 12 and 24 h [46]. Similarly, another study found significantly lower VAS scores on the first day after Milligan–Morgan hemorrhoidectomy in patients receiving BoNT [48].

The analgesic effect of BoNT extended consistently throughout the first week. One RCT demonstrated significantly lower resting pain on each of the first 7 days and during the first five defecations in the BoNT group compared to placebo [47]. Further studies confirmed this superiority at different time points later in the first week, with significant differences observed on day 3, day 5, day 6, and day 7 [48, 49]. Research also showed that the benefits of BoNT persisted up to 14 days postsurgery, with continued reductions in pain at rest and during defecation [50].

When compared to topical glyceryl trinitrate (GT) ointment, pain intensity during defecation was comparable between the two groups following Milligan–Morgan hemorrhoidectomy (*p*=0.16). However, BoNT demonstrated superior relief of resting pain over the first 7 days (*p*=0.01) [51].

For fissurectomy, BoNT injection when combined with fissurectomy yielded a higher rate of pain relief at 2 weeks postsurgery compared to fissurectomy alone (89.9% vs. 80.9%) [44]. BoNT also significantly reduced both the intensity and duration of postdefecation pain during the first postoperative defecation compared to preoperative levels (*p* < 0.0001) [45].

Overall, these findings highlight the efficacy of BoNT in reducing postoperative pain after anorectal surgeries such as hemorrhoidectomy and fissurectomy. The evidence shows that BoNT provides an additive benefit when combined with surgical interventions, such as fissurectomy, highlighting its potential as an adjunct therapy in this setting.

For pain following cholecystectomy, 72% of patients achieved at least 4 weeks of pain relief without requiring analgesics following the injection of BoNT into the sphincter of Oddi, particularly in individuals with functional biliary pain. The onset of this effect occurred at a mean of 7 days postinjection, with the pain relief lasting for a median of 8 weeks. Moreover, of the patients who initially responded to BoNT injection, 96% subsequently experienced complete pain resolution following endoscopic sphincterotomy (ES). This finding highlights the BoNT's application as a diagnostic tool for selecting ES candidates, as a safer potential alternative to sphincter of Oddi manometry (SOM) [40].

#### 3.2.3. Mastectomy and Reconstructive Surgeries

In mastectomy and reconstruction surgeries, the efficacy of BoNT for managing postoperative pain and facilitating tissue expansion demonstrates notable variability across studies. Some trials have demonstrated significant benefits. For example, one study found that BoNT significantly reduced pain scores during the immediate postoperative period, initial expansion, and final expansion after mastectomy with immediate tissue expander placement [29]. Similarly, another trial found significant pain relief between 7 and 45 days after the same surgery type with a lower dose of BoNT [39]. In contrast, other RCTs reported no measurable difference in pain relief between BoNT and placebo at any follow-up point from the first day up to 12 weeks postsurgery [37, 38].

This lack of efficacy has also been noted in other procedures, such as open abdominal wall reconstruction, where one study found no statistically significant difference in pain intensity between the treatment and control groups on any of the first five postoperative days [43]. This variability in outcomes across these studies suggests that the analgesic efficacy of BoNT is influenced by procedural factors such as dosage, injection technique, and target site. Future studies should optimize these parameters to maximize BoNT's efficacy for postoperative pain control in reconstructive surgeries.

#### 3.2.4. Head and Neck Surgeries

In head and neck surgery, BoNT demonstrated significant reductions in postoperative pain following uvulopalatopharyngoplasty (UPPP) in an RCT, in which the BoNT group experienced significantly lower overall pain intensity on postoperative days 2 and 6 (*p* < 0.01) [31]. This analgesic effect might arise from BoNT's ability to mitigate inflammation and reduce muscle tension in the pharyngeal musculature [53].

### 3.3. Analgesic Use

The evidence for BoNT's effect on analgesic consumption after hemorrhoidectomy is conflicting. While two studies reported significantly lower analgesic consumption in the BoNT group compared to control groups [47, 51], another study on the same procedure found comparable acetaminophen usage over the week following Ferguson's closed technique hemorrhoidectomy [46].

The data for mastectomy and reconstruction were more consistent, and BoNT provided both early and long-term reductions in narcotic use. One study demonstrated a significant reduction in narcotic demand on the first day after surgery and throughout the tissue expansion phases, with sustained benefits noted in both the initial and final periods [29]. Another trial reported significantly lower narcotic consumption in the BoNT group between 7 and 45 days postsurgery [39].

BoNT's effect in reducing analgesic consumption has been observed in other surgical fields. In bladder reconstruction, BoNT reduced morphine-equivalent use by more than half compared to controls [41]. Moreover, in patients undergoing UPPP, the BoNT group required significantly fewer NSAIDs during the first six postoperative days than the placebo group [31].

However, several studies have not shown this benefit in analgesic consumption. After open abdominal wall reconstruction, a study showed that total median use of morphine milligram equivalents (MME) was lower in the BoNT group (405 vs. 568.1), although the difference did not reach statistical significance (*p*=0.07) [43]. In a study on damage-control laparotomy (DCL), no significant differences were found in total MME use between the BoNT and placebo groups during hospitalization [42]. Another trial reported only slight decreases in NSAID use in the BoNT group compared to placebo following total knee arthroplasty, with no statistically significant differences in acetaminophen or MME consumption [33].

### 3.4. Complications

Table 2 provides a review of the side effects in the included studies. While BoNT injection is generally safe, it can be associated with a range of adverse effects, from minor and localized complications to systemic effects [54]. Many of these were procedure-specific complications and not directly attributable to BoNT itself. Across the reviewed studies, there were no serious side effects of BoNT, and most were mild and transient. Eight studies reported statistically comparable side effects between BoNT and control groups [33, 37–39, 41, 42, 44, 46].

#### 3.4.1. Localized Effects

Muscle weakness, a known side effect when BoNT is injected into muscles, predominantly occurs with higher doses [55]. For instance, muscle weakness was reported in two patients treated with high-dose BoNT during neck dissection surgery [30]. Similarly, four cases of transient weakness and instability were observed following intraarticular injections in knee arthroplasty patients [33]. A localized maculopapular rash was reported in the injection site in one patient, which resolved within 4 weeks [32].

#### 3.4.2. Systemic Effects

Across the reviewed studies, systemic toxicity or severe BoNT-associated adverse events were not observed, which reveals its favorable systemic safety profile. Mild systemic symptoms, potentially resulting from off-target diffusion of the toxin, were noted in several studies. For example, headaches, dry mouth, and upper respiratory issues occurred similarly in both the BoNT and control groups after total knee arthroplasty [33]. Dry mouth was also observed in two patients, receiving a high-dose BoNT injection [30]. One study on hemorrhoidectomy reported headaches only in the GT control group, not in the BoNT group [51].

#### 3.4.3. Procedure-Specific Complications

Minor transient fecal incontinence is a known issue in anal surgeries. One study reported three cases with incontinence, resolved within weeks after fissurectomy [45]. Another trial reported mild incontinence in four BoNT patients and five placebo patients, which resolved within 3 weeks [47]. However, other posthemorrhoidectomy studies observed no cases of incontinence posthemorrhoidectomy [46, 49, 52].

Bleeding was rare and comparable between the BoNT and control groups. Minor posthemorrhoidectomy bleeding was noted in a few patients receiving BoNT injections [46, 52]. Posthemorrhoidectomy urinary retention occurred significantly more in the placebo group in one study [50], while another found one case of urinary retention in each group [47].

In a cholecystectomy study, pancreatitis occurred in 15% of patients undergoing SOM but did not occur in any patients in the BoNT group [40].

Following knee arthroplasty, increased joint pain was reported in six patients in the BoNT group compared to two in the control group [33]. Conversely, after limb lengthening or deformity correction surgery, pin-site infections were significantly less frequent in the BoNT group (*p*=0.03) [35]. No difference in joint range of motion or thigh circumference was found between groups after bilateral femoral lengthening [36].

Complications such as seromas, hematomas, infection, and tissue necrosis were documented in mastectomy cases. However, these were consistent across BoNT and placebo groups, suggesting that BoNT did not exacerbate procedure-related complications [37, 38].

#### 3.4.4. Severe Outcomes

Serious adverse events were rare. One study noted while more patients in the placebo group experienced at least one serious adverse event, the difference was not statistically significant [33]. The same study also described a death from causes unrelated to BoNT, emphasizing the importance of assessing patient comorbidities. In another report, two patients of the BoNT group required readmission and reoperation for complications unrelated to the injection after open abdominal wall reconstruction [43].

## 4. Discussion

This review examines the current literature on using BoNT for postsurgical pain management. Across 24 selected studies, BoNT demonstrated effectiveness and an acceptable safety profile in this context. However, the review also revealed variability in the analgesic response, which may be attributed to several factors. Differences in demographics and baseline characteristics of the study populations, such as age, comorbidities, and type of surgery, can affect response rates. Additionally, the dosage and mode of BoNT administration, including subcutaneous, intramuscular, or intraarticular injections, influence the efficacy.

The distribution of multiple injection sites may also be a key contributor. For instance, promising results were observed when BoNT was injected into the pectoralis major, serratus anterior, and rectus abdominis during mastectomy with tissue expander placement [39]. In contrast, other studies that focused only on pectoralis major injections reported nonsignificant pain improvement [37, 38].

Although BoNT shows satisfactory analgesic effects, the timing of postinjection pain assessments influences the reported outcomes, as short-term follow-ups may fail to address the sustainability of BoNT's action. Moreover, the duration of pain relief varies across reports [56–58]. While some studies included in this review report prolonged pain relief following BoNT administration [32, 33, 39, 40], others found no evidence of long-term benefit [34–36]. This inconsistency necessitates future studies with extended follow-up periods to assess the long-term analgesic potential of BoNT.

The dose-response aspect of BoNT in pain management is critical to its therapeutic application. Generally, higher doses of BoNT are associated with lower pain intensity scores in conditions like muscular temporomandibular disorder [59]. While higher doses of BoNT may provide greater efficacy in pain relief for conditions such as lateral epicondylitis, they are also associated with a higher risk of side effects [60]. Thus, systemic side effects of BoNT, particularly muscle weakness, often necessitate the use of suboptimal doses, which can result in insufficient analgesic effects.

To address the challenge of balancing therapeutic efficacy with motor side effects, bioengineers are developing novel BoNT derivatives. One strategy is modifying the BoNT domain that targets motor neurons, substituting it with a domain that targets sensory neurons. This modification allows the resulting toxin to inhibit pain neurotransmitters, such as substance P (SP) and CGRP, while preventing muscle paralysis [61]. Another approach is to create larger toxins using methods such as SNARE-stapling. This increased molecular size limits the toxin's ability to enter the small and tight synaptic vesicles of the neuromuscular junction, thereby prohibiting local muscular weakness. This increased size does not prevent the toxin's access to open nerve endings of sensory neurons, thus maintaining its potential for pain relief [61, 62].

Ultimately, while the dose and concentration of BoNT influence its potential for pain relief, optimal dosing strategies must be further investigated to achieve the best results with the fewest side effects. For example, one study demonstrated that low doses of BoNT-A (20 U) effectively reduced chronic pain related to sleep bruxism and awake bruxism, with significant pain reduction observed up to 90 days posttreatment [63]. Similarly, when investigating the analgesic effect of BoNT for trigeminal neuralgia, both low and high doses significantly diminished pain compared to placebo, with no significant difference in efficacy between them [64]. In our review of Wittekindt et al.'s study, low-dose BoNT showed a significant improvement in VAS scores, while the high-dose regimen showed no significant impact [30].

There is limited data on the BoNT's application for postoperative pain after neurosurgical operations. A French study investigated BoNT's effectiveness in the treatment of postoperative neck pain following cervical spine surgeries, including 38 patients. Repeated injections of BoNT into the neck muscles yielded significantly better results compared to conservative treatment with muscle relaxants and physical therapy [65]. BoNT also showed benefit in one patient with intractable pain unresponsive or intolerant to a variety of conventional pharmacological and physical interventions over approximately 2 years after spinal fusion when combined with Pulsed Electromagnetic Field (PEMF) [66].

BoNT also showed promise for CPSP after craniotomy, but the evidence is mainly limited to case reports and case series. A systematic review investigated the effectiveness of BoNT for treating postcraniotomy headache (PCH). The review included five case reports and case series with a total of 15 participants who had persistent PCH. More than 70% of patients experienced a notable pain reduction and achieved 75%–100% relief within 10–15 days after the injection, without any serious complications. The patients also reported improvement in daily function and a reduced demand for pain medication. This analgesic effect lasted more than 5 years in some individuals. These results present BoNT as a potential alternative for managing refractory PCH [67]. Further larger studies are necessary to determine the optimal dosage, injection site, and number of injections for PCH.

## 5. Limitation

This review aimed to evaluate the literature on the application of BoNT for postoperative pain management. However, limitations should be considered. Our search was limited to the PubMed database, and some studies indexed in other databases might be overlooked. Most of the included studies had small sample sizes. Variations in the timing of BoNT injections, dosage, concentration, injection technique, and follow-up durations further contribute to the heterogeneity of the results. Future large, double-blind, RCT with consistent BoNT injection protocols and follow-up assessments are essential to clarify the efficacy and safety of BoNT for postoperative pain. Furthermore, high-quality systematic reviews and meta-analyses are needed to provide an analysis of the findings, considering the heterogeneity of studies.

## 6. Conclusion

The findings of this literature review emphasize the potential of BoNT injection as an effective adjunct in the management of postoperative pain in a broad spectrum of surgeries when used intra- and postoperatively. BoNT has shown promising results in reducing pain intensity and decreasing analgesic use with minimal side effects of the procedure, highlighting its safety. However, variability in outcomes between studies necessitates further randomized controlled trials with larger samples to confirm efficacy and long-term safety and optimize application protocols.

## Figures and Tables

**Table 1:** Study characteristics.

Type of surgery	Study design	Timing of injection	Sample size	Comparator	Serotype	Dosage and concentration	Method of injection	Injection site	Pain duration before injection	Follow-up timing	Pain assessment tool	Ref.
Squamous cell carcinoma-associated surgery and neck dissection	RCT	Postoperative	23 (13 in low-dose and 10 in high-dose group)	Low dose vs. high dose BoNT	A	8–12 injections of 10 MU/0.1 mL saline in low dose group and 20 MU/0.1 mL saline in high-dose group	Injections distributed equally over the pain areas at a spacing of 1.5 cm	The target area was determined based on physical examination	After 1.1 ± 0.4 years of pain	Days 0 and 28	VAS	[30]
UPPP	RCT	Intraoperative	58 (31 in the BoNT group and 27 in the control group)	Placebo (0.2 mL saline)	A	10 IU, 5 IU injected into the uvula and 5 IU applied to the tonsillectomy region using a cotton ball	Intramuscular and topical application	Upper region of the uvula and tonsillectomy region	N/A	Days 2 and 6	5-point scale (0 = no pain, 4 = severe pain)	[31]
Patients with cancer after surgery or/and radiation therapy	Prospective, open-label, single-arm pilot study	Postoperative	12 (8 completed; all undergone surgery ± radiation)	N/A	A	80–100 U in normal saline (100 units per mL)	Subcutaneous or intramuscular	Depending on pain location	N/M	Weeks 4, 6, 8 and 12	VAS	[32]
Total knee arthroplasty for more than 6 previous months	RCT	Postoperative	60 (30 cases and 30 controls)	Placebo (5 mL saline)	A	100 U in 5 mL normal saline (100 units/5 mL) in case group 5 mL normal saline in control group	Intraarticular via standardized lateral or medial method	Knee joint	N/M	Week 2, months 1, 2, 3, and 4	VAS	[33]
Limb lengthening	RCT	Intraoperative	36	Saline with similar amount injected into the other leg	A	200 U at 20 mL normal saline in one leg	Intramuscular	At six different locations of gastrocnemius and soleus muscles	N/A	Minimum of 24 months	VAS	[34]
Limb lengthening or deformity correction	RCT	Intraoperative	125 (62 cases and 63 controls)	Saline	A	10 U/kg, max 400 U in 1 mL normal saline	Intramuscular	Quadriceps, hamstrings, and gastrocnemius-soleus	N/A	First 4 postop days	NRS for > 12 years, faces pain scale for < 12 years, APPT	[35]
Bilateral femoral lengthening for familial short stature	RCT	Intraoperative	44 participants and a total of 88 femoral segments	Placebo (saline into another thigh)	A	200 U in 20 mL of sterile saline	Intramuscular	Seven locations across the anterior thigh muscles of rectus femoris, sartorius, tensor fascia lata, and vastus muscles	N/A	Weekly during the distraction phase and monthly until the end of the 48-week consolidation phase	VAS	[36]
Bilateral mastectomy with tissue expander reconstruction	RCT	Intraoperative	23 patients receiving BoNT or placebo in each breast	Placebo (saline)	N/M	100 U in 10 mL saline	Intramuscular	Pectoralis major muscle	N/A	Postoperative day 1, and weeks 1–2, 3–4, 5–6, 7–8, 9–10, 11–12, and beyond	VAS	[37]
Mastectomy and immediate subpectoral tissue expander breast reconstruction	RCT	Intraoperative	131 (68 cases and 63 controls)	5 mL normal saline placebo	A	100 U in 5 mL normal saline	Intramuscular	Pectoralis major muscle	N/A	Preop, first postop visit, at every expansion visit, and final plastic surgery visit	Numeric pain scale	[38]
Mastectomy with tissue expander placement	Nonrandomized prospective clinical trial	Intraoperative	48 (22 cases and 26 controls)	Placebo	A	100 U in 40–60 mL normal saline	Intramuscular	Pectoralis major, serratus anterior, and rectus abdominis insertion	N/A	Immediate postsurgical and during initial and final expansion pain	VAS	[29]
Mastectomy with immediate expander or acellular dermal matrix reconstruction	RCT	Intraoperative	30 (15 cases and 15 controls)	4 serial injections of 2cc saline	A	40 U in 2cc saline into each pectoralis major muscle	Four serial intramuscular injections	Pectoralis major	N/A	1 year	VAS	[39]
Cholecystectomy	Retrospective observational study	Postoperative	64	41 patients received both SO manometry and BoNT injections	A	100 U in 2 mL normal saline	Endoscopic using a 23G variceal injection needle	Injected into four ampulla quadrants of sphincter of Oddi	Frequent episodic biliary pain with at least 4 monthly episodes	At least 4 months for BoNT group after sphincterotomy	Patient self-reported episodic biliary pain relief	[40]
Open continent bladder reconstruction	Retrospective cohort study	Intraoperative	30 (15 cases and 15 controls)	No BoNT	A	200–300 U (10 U/1cc)	*Trans*-urothelial intravesical	Bladder wall	N/A	Postop pain and medication usage evaluated during hospitalization	—	[41]
DCL for trauma and emergency general surgery	Double-blind, placebo-controlled, randomized pilot study	Postoperative	46 patients (24 in the BoNT group and 22 in the placebo group)	Placebo (150 mL saline)	A	150 U in 300 mL normal saline (2 U/mL)	Intramuscular and ultrasound-guided	External oblique, internal oblique, and transversus abdominis muscles bilaterally, with six total injection sites (right/left subcostal, anterior axillary, and lower quadrants)	N/A	Daily for the first 5 days; complications and outcomes were monitored during the hospital stay	Morphine equivalent use	[42]
Open abdominal wall reconstruction with retrorectus release	Retrospective matched case series	Intraoperative	41 patients (19 in the BoNT group and 22 in the placebo)	No BoNT	A	200 U in 30 mL saline	Intramuscular	Bilateral oblique, transversus, and rectus muscles	N/A	Day 0–5, and outpatient follow-up within 50 days	VAS	[43]
Fissurectomy	Retrospective	Intraoperative	293 (168 fissurectomy + BoNT, 115 fissurectomy)	Fissurectomy without BoNT injection	A	20–50 U	Intersphincteric, divided into quadrants	Internal anal sphincter	Persistent anal pain > 8 weeks before surgery	First visit at 14 days postop	Patient-reported pain relief	[44]
Fissurectomy with anoplasty	Prospective pilot single-arm study	Intraoperative	10 patients	No comparator	A	30 U, in saline (50 units/mL)	Intersphincteric	Internal anal sphincter on both sides of the posterior midline	N/A	Months 1, 6, and 12	VAS	[45]
Ferguson hemorrhoidectomy	RCT	Intraoperatives vs. preoperative	62 patients (31 in the early BoNT group and 31 in the intraoperative BoNT group)	Early BoNT injection (7 days before surgery) vs. intraoperative BoNT injection	A	50 U in 3 mL of distilled water	Intersphincteric injection of 25 U bilaterally	Bilateral lateral aspects of the internal anal sphincter	N/A	Days 0–6, with outpatient follow-up until clinical wound healing	VAS	[52]
Ferguson's closed technique hemorrhoidectomy	RCT	Intraoperative	82 patients (39 in the BoNT group and 43 in the control group)	No injection group	A	30 U in 0.5 mL of solution	Intersphincteric immediately after excision of hemorrhoids	Intersphincteric plane near the widest excised hemorrhoid site	N/A	Hours 12- and 24-h postsurgery; follow-up data collected at weeks 2, 4, and 16	VAS	[46]
Hemorrhoidectomy	RCT	Intraoperative	30 (15 cases and 15 controls)	Placebo (0.4 mL of saline)	A	0.4 mL of solution containing 20 units BoNT in case group (50 U/mL)	Two intersphincteric injections	Internal anal sphincter	N/A	Rest pain 3 times daily for 1 week, Defecation pain up to the 5^th^ defecation	linear analog 0 to 10 scale	[47]
Milligan–Morgan hemorrhoidectomy	RCT	Intraoperative	40 patients (20 in the BoNT group and 20 in the control group)	No injection group	A	150 U in 0.5 mL solution	Intersphincteric immediately after hemorrhoidectomy	Left and right lateral intersphincteric areas	N/A	Days 1, 3, 5, and 7	VAS	[48]
Milligan–Morgan hemorrhoidectomy	RCT	Intraoperative	50 (25 in each group)	Placebo (saline)	A	20 U in 0.4 mL (50 U/mL)	Two intersphincteric injections	Internal sphincter	N/A	Pain diaries completed daily for the first 7 days; follow-up after 1 and 6 weeks	VAS	[49]
Milligan–Morgan hemorrhoidectomy	RCT	Intraoperative	67 patients (34 in the BoNT group and 33 in the placebo group)	Placebo (saline)	A	20 U in 0.4 mL (50 units/mL)	Intersphincteric	Internal anal sphincter at two points in the anterior midline	N/A	Hours 6, 12, 24, and 48, and on days 7 and 14	VAS	[50]
Milligan–Morgan hemorrhoidectomy	RCT	Intraoperative	30 (15 BoNT 15 GT)	300 mg topical GT ointment 0.2% three times daily for 1 month	A	20 IU in 0.4 mL with a concentration of 50 IU/mL	Two injections on each side of the sphincter, localized in the anterior midline	Internal sphincter	N/A	Rest pain three times daily for 1 week; defecation pain up to the 5^th^ defecation	VAS	[51]

*Note:* BoNT = botulinum neurotoxin, SO = sphincter of Oddi, UPPP = uvulopalatopharyngoplasty.

Abbreviations: APPT = adolescent pediatric pain tool, DCL = damage-control laparotomy, GT = glyceryl trinitrate, IU = international unit, NRS = numeric rating Scale, RCT = randomized controlled trial, VAS = visual analog scale.

**Table 2:** Effect of BoNT on postoperative pain reduction, analgesic consumption, and associated complications.

Pain-related outcomes	Complications	Ref.
A significant reduction in VAS scores was observed in the low-dose group (from 4.3 ± 1.0 on day 0–3.0 ± 1.9 on day 28, *p* < 0.05). No significant pain improvement was seen in high-dose group (from 4.2 ± 1.5 on day 0–4.3 ± 3.3 on day 28, *p*=0.86)	No serious complications were observed. Muscle weakness occurred in two patients of the high-dose group	[30]
Lower pain intensity was observed in the BoNT group compared to the control group (3.61 vs. 3.45 on day 2 and 0.3 vs. 2.08 on day 6, *p* < 0.01). Fewer NSAIDs was required in the BoNT group during the first 6 postsurgical days (4.14 NSAID pills compared to 9.26 pills in the control group, *p* < 0.001)	The BoNT group experienced less foreign body sensation in the throat (0.58 vs. 1.48, *p* < 0.01). Postoperative bleeding occurred in two patients in the BoNT group and three patients in the control group, all managed without major interventions. Dry mouth was reported in two patients in the BoTN group and one patient in placebo group. No cases of velopharyngeal insufficiency were observed	[31]
All patients experienced significant pain reduction in the 6 weeks follow-up. Five of the eight patients maintained this improvement at the 12 weeks. At both 6 and 12 weeks, all patients stated some reduction in pain, with seven out of eight indicating they felt much or very much better	A maculopapular rash was observed in one patient on her upper back shortly after the injection, faded over 4 weeks; however, a cause-and-effect link could not be determined	[32]
Higher responder rates were observed in the BoNT group at 2 months (71 vs. 35, *p*=0.025) and at all three time points of 2, 3, and 4 months (*p*=0.019). The BoNT group experienced a significantly longer duration of meaningful pain relief (39.6 days) compared to the placebo group (15.7 days; *p*=0.045) with the onset of meaningful pain relief in the BoNT group at 66.5 days minimal changes in analgesic intake were noted at the 2-month follow-up, with a slight decrease in the NSAID dose of the BoNT group (0.1 ± 0.32 DDD), versus an increase in the placebo group (0.02 ± 0.29 DDD, *p*=0.21). No significant differences were observed between the groups in acetaminophen (60 mg decrease vs. 186 mg decrease, *p*=0.71) or MME doses (0.66 increase vs. 0.50 decrease; *p*=0.67)	Adverse events were comparable between BoNT and placebo groups (66 in BoNT vs. 73 in controls, *p*=0.76), as were serious complications (5 vs. 11, *p*=0.47). More patients experienced at least one serious adverse event in the placebo group (9 vs. 3, *p*=0.16). One death in the BoNT group (a 64-year-old woman with several comorbidities) was unrelated to the injection. Increased joint pain occurred in 6 BoNT vs. 2 placebo patients; transient knee weakness in 4 BoNT vs. 2 placebo. No new motor or sensory deficits or joint inflammation were observed. Systemic adverse events were similar, including dry mouth (4 BoNT vs. 3 controls), upper respiratory issues (10 in each group), accidental injuries (11 vs. 7), headaches (3 vs. 2), chest pain (0 vs. 3), and back pain (3 vs. 4). Most events were linked to preexisting medical conditions or treatments, not the injection	[33]
There was no significant difference in pain intensity between the groups at 6 weeks (3 [0–8] vs. 3 [0–10], *p*=0.61), 12 weeks (3 [0–7] vs. 3 [0–7], *p*=0.53), 24 weeks (1 [0–4] vs. 1 [0–3], *p*=0.91), and 48 weeks (38 [30–41] vs. 38 [30–42], *p*=0.85) following the procedure	N/M	[34]
The BoNT group had significantly lower maximum postoperative pain scores on day 1 compared to placebo (4.0 vs. 5.3, *p*=0.03). No significant differences were observed beyond day 1 (day 2: mean 4.42 in BoNT vs. 4.26 in control; day 3: 3.64 in BoNT vs. 3.38 in control; day 4: 2.62 in BoNT vs. 3.59 in control)	No severe complications were attributed to BoNT. Adverse effects occurred in 66% of the BoNT group vs. 76% of controls. Pin-site infections were less frequent in the BoNT group (40% vs. 51%, *p*=0.03).	[35]
There was no significant difference in pain scores between the BoNT group and the placebo group throughout the study period at postoperative weeks 4 (3.0 ± 2.2 vs. 4.0 ± 3.0, *p*=0.09), 8 (1.6 ± 1.9 vs. 1.8 ± 2.2, *p*=0.60), 12 (1.3 ± 1.6 vs. 1.5 ± 1.6, *p*=0.70), 24 (0.6 ± 1.9 vs. 0.1 ± 0.9, *p*=0.16), and 48 (0.1 ± 0.8 vs. 0.6 ± 1.9, *p*=0.14)	No adverse effects were associated with BoNT injection. There was no difference in joint range of motion or thigh circumference between groups	[36]
There was no measurable difference in pain relief between BoNT and placebo sides at any time point. The mean pain intensity changes from preoperative levels were: day 1: 4.89 (2.80) in the BoNT group vs. 4.13 (1.86) in the placebo group, *p*=0.22; weeks 1–2: 2.69 (2.06) vs. 2.31 (1.50), *p*=0.45; weeks 3–4: 1.70 (2.14) vs. 1.43 (1.90), *p*=0.40; weeks 5–6: 0.58 (1.58) vs. 0.87 (1.53), *p*=0.49, weeks 7–8: 0.33 (1.50) vs. 0.03 (1.27), *p*=0.40; weeks 9–10: 0.67 (1.24) vs. 0.12 (1.40), *p*=0.08; weeks 11–12: 0.60 (1.26) vs. 0.20 (1.57), *p*=0.15; weeks > 12: 0.17 (0.98) vs. 0.33 (0.82), *p*=0.36	Complications occurred in 28% of implants, including seromas (2 BoNT and 1 placebo), hematomas (2 BoNT), infections (2 BoNT and 3 placebo), expander ruptures (1 placebo), and skin necrosis (2 BoNT and 1 placebo). There was no significant difference between BoNT and placebo groups (*p*=0.53)	[37]
No meaningful changes in pain were observed in the BoNT group compared to placebo relative to the preoperative pain intensity at the first postoperative visit (0.22 [IQR, 0.08–0.43] in BoNT vs. 0.29 [IQR, 0.14–0.46] in placebo, *p*=0.18) or throughout tissue expansion (−0.01 [IQR, −0.02 to 0.00] BoNT vs. −0.01 [IQR, −0.02 to 0.00] placebo, *p*=0.55)	No adverse events were linked to BoNT injection. There was no significant differences in the complication rates between groups, including seroma (0% BoNT vs. 6% placebo, *p*=0.051), hematoma (1% BoNT vs. 3% placebo, *p*=0.61), infection (1% BoNT vs. 3% placebo, *p*=0.61), flap necrosis (1% BoNT vs. 0% placebo, *p*=1.0), reconstruction failure (1% BoNT vs. 0% placebo, *p*=1.0), and delayed wound healing (1% BoNT vs. 3% placebo, *p*=0.44)	[38]
Significant reduction in pain scores was observed during immediate postoperative period (3 ± 1 vs. 7 ± 2), initial expansion (2 ± 2 vs. 6 ± 3, *p* < 0.00001), and final expansion (1 ± 1 vs. 3 ± 2, *p*=0.009). The control group had significantly higher narcotic demand during the first day (17 ± 10 mg vs. 3 ± 3 mg, *p* < 0.0001), initial expansion (*p*=0.012), and final expansion period (*p*=0.037)	No complications were observed with BoNT. Lower expander removal rates were seen in the BoNT group (1 vs. 5, *p*=0.13)	[29]
The BoNT group experienced significantly lower pain intensity from day 7 to day 45 (*p* < 0.05). Narcotic use was comparable between two groups in the first three postoperative days (*p*=0.59–0.79); however, narcotic use significantly reduced in the BoNT group on 7–45 days after the procedure (*p* < 0.05)	No complications were related to the BoNT administration. No substantial differences were observed in the frequency of complications between the two groups	[39]
At least 4 weeks of pain relief without the need for analgesics were observed in 72% of patients. Pain relief began after an average of 7 days, with all responsive patients noting relief within 14 days. The median duration of pain relief was 8 weeks (range 4–14 weeks), with pain returning within 16 weeks for all responders	There were no complications in the BoNT group. Pancreatitis occurred in 15% of the SOM group, and one required 7-day hospitalization	[40]
BoNT group used significantly less postsurgical narcotic medication compared to the control group (0.32 vs. 0.85 morphine mEq/kg/day, *p*=0.0002)	Complications occurred similarly in both groups (overall: 6 in BoNT vs. 5 in controls, *p* > 0.99; UTI: 2 vs. 0, *p*=0.48; urine leak: 1 vs. 0, *p*=0.33; hematuria requiring irrigation or clot evacuation: 0 vs. 0, *p* > 0.99; superficial surgical site infection: 1 vs. 2, *p*=0.56; C. difficile infection: 1 vs. 1, *p* > 0.99; ureteral obstruction requiring nephrostomy tube: 1 vs. 1, *p* > 0.99; gastrointestinal bleed: 0 vs. 1, *p*=0.33)	[41]
Both groups showed similar MME use across all five postoperative days, with median (interquartile range) values as follows: day 1: 112 (63.4–147.3) in BoNT vs. 90.8 (44.6–144.8) in placebo, *p*=0.13; day 2: 105 (67.3–157.8) vs. 86.2 (40–155.5), *p*=0.21; day 3: 88.9 (52.3–153.6) vs. 95.3 (38.8–120.1), *p*=0.37; day 4: 55.5 (30.7–148.7) vs. 80.9 (33.8–99.4), *p*=0.40; day 5: 53.8 (23.0–136.2 vs. 45.5 (25.6–64.5), *p*=0.19	Overall complication rates were comparable between groups (63% in the BoNT group vs. 68% in the placebo group, *p*=0.69). No complication were attributed to BoNT injection	[42]
Both groups showed no significant differences in pain scores from day 0–5 (day 0: median: 3.2 in BoNT vs. 7.5 in control, *p*=0.16; day 1: 5.4 vs. 6.4, *p*=0.46; day 2: 3.7 vs. 6.9, *p*=0.27; day 3: 3.1 vs. 6.3, *p*=0.77; day 4: 4.3 vs. 6.7, *p*=0.40; day 5: 3.5 vs. 5.7, *p*=0.72). Total median MME use was lower in the BoNT group compared to the no-BoNT group (405 vs. 568.1, *p*=0.07)	No adverse events were related to BoNT. Readmission occurred in two patients in each group (*p*=1) and a return to the operating room in two patients in the BoNT group vs. 0 of the controls (*p*=0.21) due to complications unrelated to the injection	[43]
Pain relief rate increased from 80.9% in fissurectomy alone group to 89.9% in patients with BoNT (*p* < 0.05)	Adverse effects were similar between groups: incontinence (4.2%), bleeding (2.1%), infection (1.4%). No complications occurred due to BoNT injection itself	[44]
Postdefecation pain intensity (3.3 ± 1.1) and duration (29.5 ± 15.3) were significantly reduced during the first defecation after surgery compared to the preoperative values (*p* < 0.0001). Complete healing was achieved within 30 days in all patients	Minor postoperative complications included two donor site infections and one partial wound breakdown. Temporary anal incontinence occurred which resolved in all but one patient by 12 months. No recurrences were observed during the follow-up	[45]
A Resting pain intensity was significantly lower in the early BoNT (preoperative) group compared to the intraoperative group from postoperative days 1–5 (day 0: 5.8 ± 2.5 vs. 6.8 ± 2.1, *p*=0.057; day 1: 4.5 ± 2.6 vs. 6.0 ± 2.4, *p*=0.014; day 2: 4.5 ± 2.4 vs. 5.3 ± 2.1, *p*=0.025; day 3: 4.7 ± 2.4 vs. 5.7 ± 2.3, *p*=0.014; day 4: 4.4 ± 2.3 vs. 5.5 ± 2.1, *p* < 0.001; day 5: 4.4 ± 2.4 vs. 5.1 ± 1.9, *p*=0.008). By day 6 the difference was no longer statistically significant (4.3 ± 2.7 vs. 4.8 ± 2.2, *p*=0.063)	Anal bleeding occurred in one patient in the intraoperative BoNT group on postoperative day 6, with no cases of incontinence in either group	[52]
Pain intensity was significantly lower in the BoNT group compared to the control group at both 12 h (4.4 ± 2.1 vs. 6.2 ± 2.3; *p* < 0.001) and 24 h (2.2 ± 2.1 vs. 3.7 ± 2.4; *p*=0.003) postsurgery. Acetaminophen use during the first week was similar between groups, with a median (min-max) of 4000.0 mg (0.0–10000.0) vs. 4000.0 mg (0.0–10000.0), *p*=0.881	Early and late complications did not differ significantly between groups. Minor postoperative bleeding occurred in two patients in the BoNT group. No cases of incontinence during follow-up	[46]
Postoperative resting pain was significantly reduced overall (*p* < 0.0001) and on each of the seven postoperative days (day1: 3.9 BoNT vs. 5.98 control, *p* < 0.0001; day2: 3.27 vs. 7.3, *p* < 0.0001, day 3: 3.45 vs. 7, *p* < 0.0001; day 4: 3.07 vs. 5.65, *p* < 0.0001, day 5: 2.81 vs. 5.11, *p* < 0 0.0001; day 6: 2.5 vs. 3.34, *p* < 0.0001). Pain during defecation was also significantly lower in BoNT group compared to placebo overall (*p* < 0.0001) and at all five follow-ups (first: 4.05 vs. 7.32, *p* < 0.0001; fifth: 2.5 vs. 3.34, *p*=0.0029). The placebo group also used more analgesic tablets than the BoNT group (22.3 ± 5.1 vs. 14.8 ± 6.2, *p* < 0.05)	No severe complications were observed. Mild incontinence occurred in four BoNT patients and five placebo patients, resolving within 3 weeks. Urinary retention was reported in one patient from each group. No bleeding, stenosis, or BoNT-related effects were noted	[47]
Pain scores were significant lower in the BoNT group compared to the control group at three time points: day 1 (7.6 vs. 8.25, *p*=0.054); day 3 (4.05 vs. 6.05, *p* < 0.001), and day 5 (2.45 vs. 3.05, *p*=0.006), while the control group showed lower pain on day 7 (2.05 vs. 1.7, *p*=0.031). During the first defecation, the experimental group had a mean VAS score of 2.70 ± 1.17 compared to 6.45 ± 1.23 in the placebo group (*p* < 0.001)	Gas incontinence prevalence was not assessed due to the rarity and overlap with surgical side effects	[48]
The BoNT group had significantly lower pain scores on days 6 and 7 compared to placebo (*p*=0.02 and 0.04, respectively)	No complications were reported. Patients in both groups had no fecal incontinence	[49]
Pain at rest significantly reduced in the BoNT group at 12 h (3.4 vs. 4.5), 24 (2.3 vs. 3.8), and 48 h (1.12 vs. 3.3), as well as on days 7 (0.4 vs. 2.4), and 14 (0.3 vs. 1.3) (*p* < 0.01). Pain during defecation was also lower in the BoNT group from the 3rd to 5th defecation (*p* < 0.01)	No adverse effects were observed in the BoNT group. The placebo group experienced significantly more frequent urinary retention (0 vs. 5, *p*=0.02) and severe itching (0 vs. 6, *p*=0.01)	[50]
A significant pain reduction at rest was observed in the BoNT group compared to the GT group at 7 days (*p*=0.01) despite similar overall pain during first five defecations in both groups (*p*=0.16). The GT group also used a greater number of analgesic tablets than the BoNT group (20.4 ± 6.1 vs. 16.8 ± 5.3; *p* < 0.05)	Only minimal side effects were reported, including mild temporary incontinence in five patients in GT group vs. four in the BoNT which resolved within 3 weeks without significant differences. Adverse effects possibly associated with drug administration were significant exclusively in the GT group (*p* < 0.03). Headaches occurred only in the GT group	[51]

*Note:* BoNT: botulinum neurotoxin, C. difficile: *Clostridium difficile*, Ref.: Reference.

Abbreviations: DDD = defined daily dose, GT = glyceryl trinitrate, MME = morphine milligram equivalent, N/M = not mentioned, NSAID = nonsteroidal anti-inflammatory drug, SOM = sphincter of Oddi manometry, UTI = urinary tract infection, VAS = visual analog scale.

## Data Availability

Data sharing is not applicable to this article as no datasets were generated or analyzed during the current study.

## Supporting Information

The detailed PubMed search strategy is provided in Supporting Table 1. Additionally, two other studies were retrieved through manual searching.
