# Supplementary material for: Intra- and Postoperative Botulinum Toxin Injection in Postsurgical Pain Management: A Literature Review
Source: Pain Res Manag. 2025 Nov 30;2025:6649252. doi: 10.1155/prm/6649252 (PMC12682448; doi:10.1155/prm/6649252)
Supplement: Supporting Information — Additional supporting information can be found online in the Supporting Information section. [file 6649252.f1.docx]

**Supplementary Table 1.** Search strategy

| 1. **PubMed:** **248** (13 January 2025) |
| --- |
| ("pain, postoperative"[MeSH Terms] OR ("pain"[All Fields] AND "postoperative"[All Fields]) OR "postoperative pain"[All Fields] OR ("postoperative"[All Fields] AND ("Pain" OR "Discomfort"[All Fields] OR "Aching"[All Fields])) OR "postsurgical pain"[All Fields] OR "surgical pain"[All Fields] OR "pain after surgery"[All Fields] OR "postoperative discomfort"[All Fields] OR "postoperative analgesia"[All Fields] OR "acute postoperative pain"[All Fields] OR "chronic postoperative pain"[All Fields]) AND ("botulinum toxins"[MeSH Terms] OR ("botulinum"[All Fields] AND "toxins"[All Fields]) OR "botulinum toxins"[All Fields] OR ("botulinum"[All Fields] AND "toxin"[All Fields]) OR "botulinum toxin"[All Fields] OR "BTX"[All Fields] OR ("botulinum"[All Fields] AND "neurotoxins"[All Fields]) OR "botulinum neurotoxins"[All Fields] OR ("clostridium"[All Fields] AND "botulinum"[All Fields] AND "toxins"[All Fields]) OR "clostridium botulinum toxins"[All Fields] OR "botulin"[All Fields] OR "botulinic"[All Fields]) |
| **2. Other sources (searching manually): 2** |
